# Clinical and functional significance of a novel ferroptosis‐related prognosis signature in lung adenocarcinoma

**DOI:** 10.1002/ctm2.364

**Published:** 2021-03-17

**Authors:** Ziqi Wang, Jie Diao, Xingru Zhao, Zhiwei Xu, Xiaoju Zhang

**Affiliations:** ^1^ Department of Respiratory and Critical Care Medicine Zhengzhou University People's Hospital, Henan Provincial People's Hospital Zhengzhou Henan China; ^2^ Department of Electronics and Electrical Engineering and Management The University of Glasgow Scotland UK; ^3^ Clinical Research Service Center Henan Provincial People's Hospital, Zhengzhou University People's Hospital, Zhengzhou Henan China

**Abbreviations**
ACSL3: Acyl‐CoA Synthetase Long Chain Family Member 3
ALOX15: Arachidonate 15‐Lipoxygenase
AUC: area under curve
DEGs: differential expressed genes
DPP4: Dipeptidyl Peptidase 4
GCLC: glutamate‐cysteine ligase catalytic subunit
GO: gene ontology
KEGG: Kyoto Encyclopedia of Genes and Genomes
KM‐plot: Kaplan Meier‐plot
KRAS: kirsten rat sarcoma viral oncogene
LUAD: lung adenocarcinoma
MAP1LC3C: Microtubule Associated Protein 1 Light Chain 3 Gamma
MCA: multivariate cox analysis
NCOA4: nuclear receptor coactivator 4
NRAS: neuroblastoma RAS viral oncogene homolog
OS: overall survival
SLC11A2: Solute Carrier Family 11 Member 2
SLC3A2: Solute Carrier Family 3 Member 2
SLC7A11: Solute Carrier Family 7 Member 11
ssGSEA: single sample gene set enrichment analysis
TCGA: The Cancer Genome Atlas
TFs: transcription factors
TP53: Tumor Protein P53
tROCs: time dependent receiver operating curves
UCA: univariate cox analysis
VDAC2: Voltage Dependent Anion Channel 2
VIF: variance inflation factor

Dear Editor,

Lung cancer remains the most common and fatal type of cancer around the world, and lung adenocarcinoma (LUAD) is the most common subtype.^1^ Although gene‐targeted therapies and immunotherapies have made great advances, most patients still suffer from drug‐resistance or insensitivity to current regimens.^2^ Ferroptosis is a new regulated cell death form defined in 2012,^3^ recently, its relevance in multiple pathological process including malignant tumors^4^ has been reported, which is of great prognostic and therapeutic interests. Here, we aim to identify novel prognostic genes and construct a ferroptosis‐related prognostic signature for LUAD.

As shown in the flow chart (Figure 1A), RNA‐seq data of LUAD were retrospectively downloaded from The Cancer Genome Atlas (TCGA) database as discovery cohort, which contained 505 tumor tissues and 59 normal lung tissues after exclusion of samples without clinical or survival information. GSE72094 datasets were obtained from gene expression omnibus, and a total of 398 cases were used as an independent validation cohort. Ferroptosis‐related genes were selected based on the ferroptosis database (http://www.zhounan.org/ferrdb/) and literature review (Table S1). Differential analysis in discovery cohort showed that most of the genes (194/274, 70.08%) were abnormally expressed in LUAD (Figure 1B). Univariate cox analysis (UCA) revealed 49 prognostic genes (49/274, 17.89%, Figure 1C), of which KRAS, NRAS, and SLC7A11 maybe hub proteins in protein‐protein interaction network (Figure 1D). Following least absolute shrinkage and selection operator identified 10 genes (Figures 1E and 1F), proportional hazards (PH) test and variance inflation factor (VIF) were then used to ensure the time‐independence and non‐collinearity of these genes. MAP1LC3C (*p* = 0.00017) and SLC7A11(*p* = 0.0093) were excluded due to failure on PH test, finally eight genes with a *p*‐value larger than 0.05 on PH test and a VIF smaller than 2 were selected for modeling using Cox model (Table S2), and risk score was as follow: (3.40*expression of ACSL3)+ (−0.32*expression of ALOX15)+ (0.16*expression level of DPP4)+ (0.14*expression of GCLC)+ (−3.93*expression of NCOA4)+ (−3.00*expression of SLC11A2)+ (0.05*expression of SLC3A2)+ (2.45*expression of VDAC2). Waterfall map showed that the somatic alteration rates vary from 1% to 3% among eight genes (Figure 2A). Expression of ALOX15 was significantly decreased in tumor in TCGA (Figure S2A). Sample‐matched analysis showed that except for SLC11A2, all genes were abnormally expressed in tumor tissues (Figure 2B). In addition, VDAC2 was increasingly expressed along with disease progression (Figure S2B).

**FIGURE 1:**
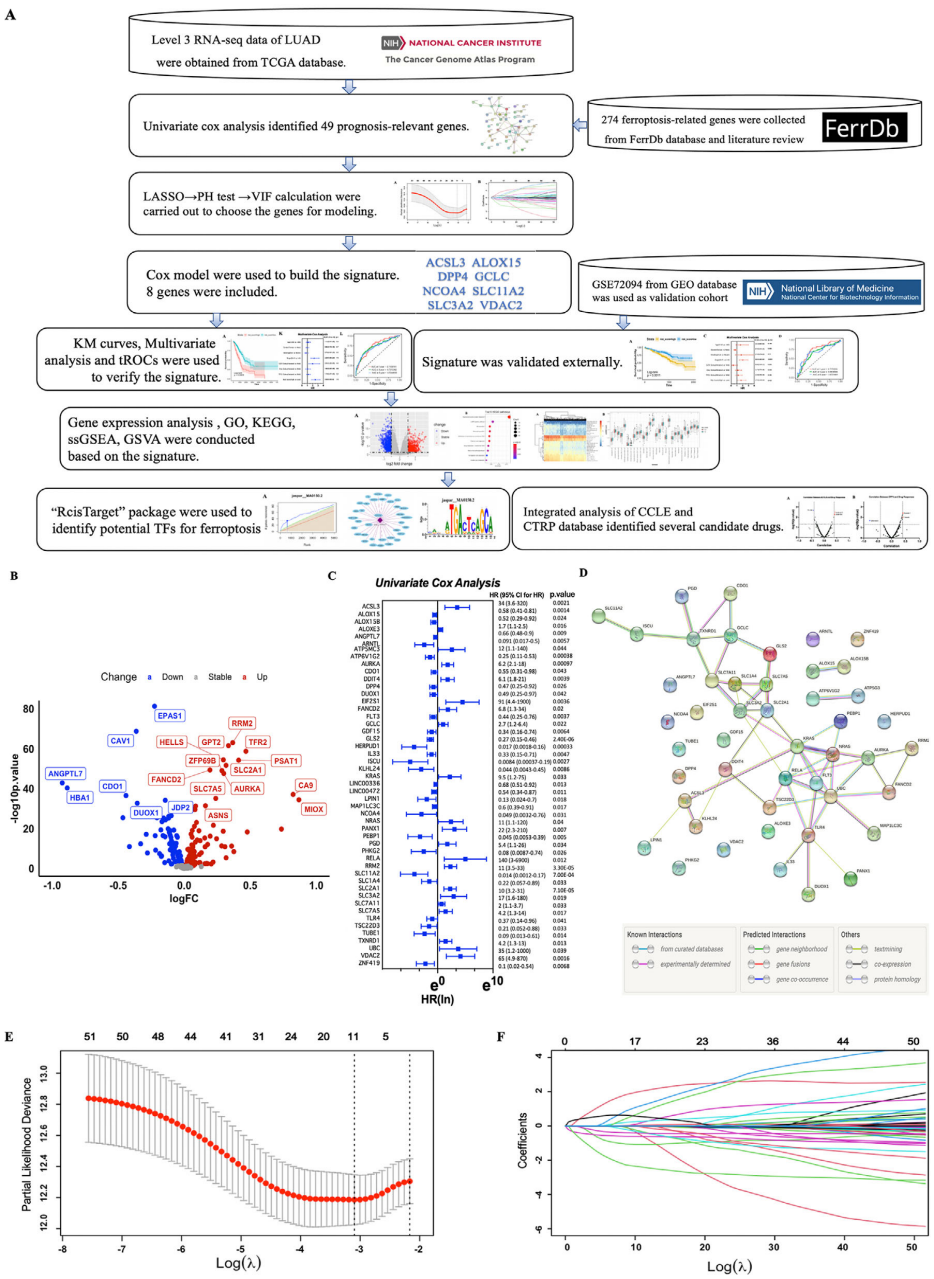
(A) Flow chart of bioinformatic analysis. (B) Differentially expressed ferroptosis‐related genes between tumor and adjacent normal tissue in TCGA database. (C) Forrest plot demonstrates that a total of 49 ferroptosis‐related genes were identified as prognosis related by univariate cox analysis(*p* < 0.05). (D) PPI network showing interaction among proteins coded by those genes. (E) LASSO regression with tenfold cross‐validation. (F) LASSO coefficients profiles of 49 prognosis‐related genes. Abbreviations: LASSO, least absolute shrinkage and selection operator; PPI, protein‐protein interaction, TCGA, The Cancer Genome Atlas

**FIGURE 2:**
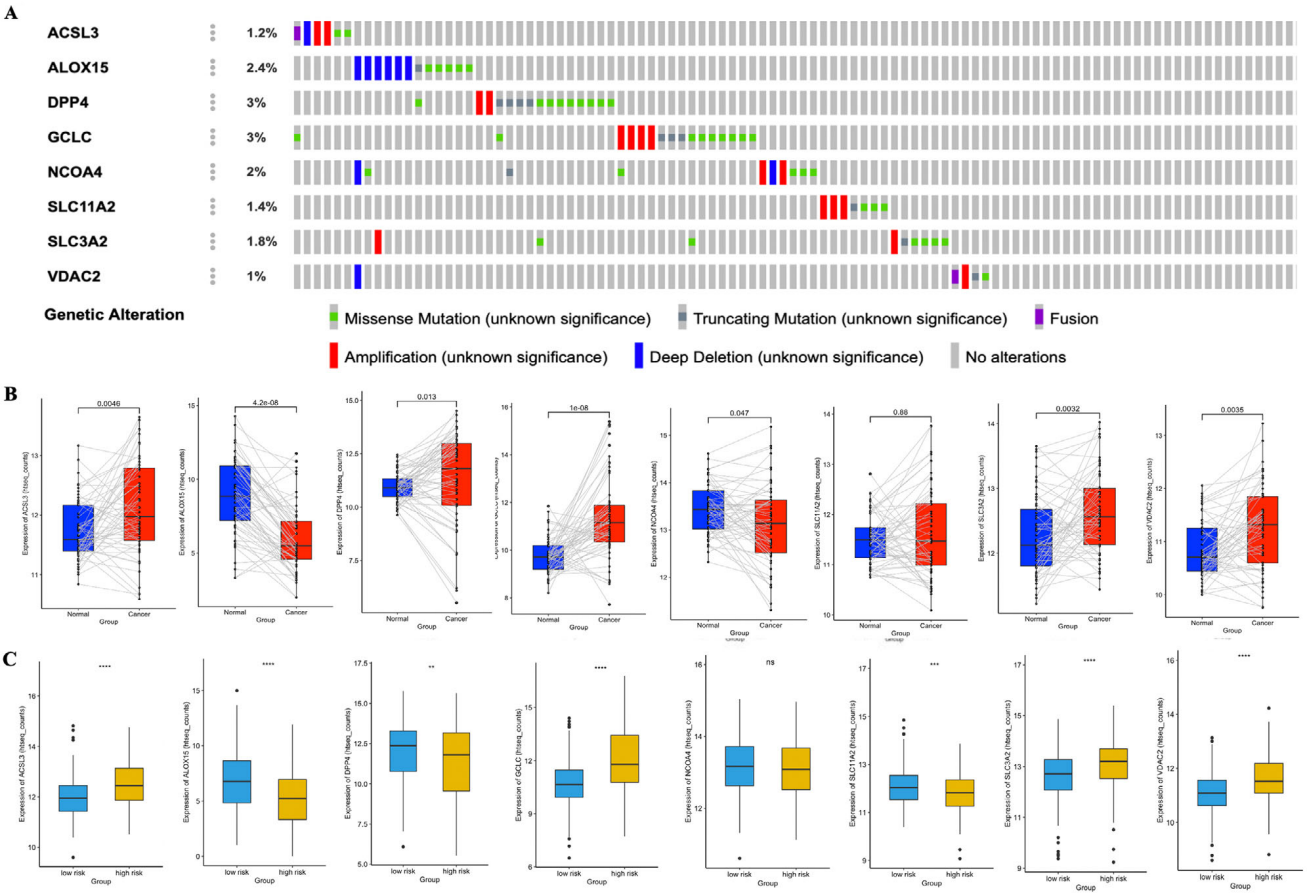
(A) Waterfall map of somatic alteration of eight hub genes from cBioPortal, each grey bar represents one patient. Data of patients without alteration were partly truncated due to page space limitation. (B) Sample‐matched analysis with Wilcox‐test. (C) Differential analysis between low‐ and high‐risk group. *p < 0.05, **p < 0.01, ***p < 0.001. Abbreviation: ns, not significant

Patients were then divided into high‐ or low‐risk group according to mean value of risk score. TP53 and KRAS, which were reported to be closely related with ferroptosis,^5^ were more frequently mutated in high‐risk group (Table S3). Kaplan Meier (KM)‐plot showed that patients in high‐risk group correlated with a significantly worse overall survival (OS), no matter in overall population (Figure 3A) or subgroup analysis (Figure S1). UCA showed that stage, EGFR mutation status, and risk score were prognosis‐related (Figure 3B); multivariate cox analysis (MCA) revealed that stage and risk score were independent predictive factors for OS (Figure 3C). Time dependent receiver operating curves (tROCs) showed that risk score has a maximum area under curve (AUC) of 0.7439144 at 1‐year (Figure 3D).

**FIGURE 3:**
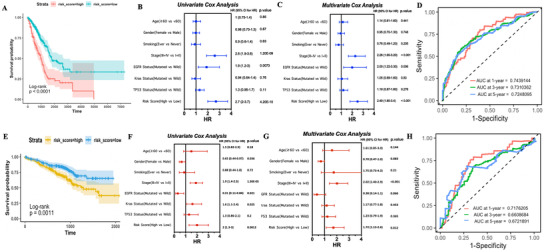
(A‐D) Prognosis analysis of eight‐genes signature in TCGA. (E‐H) Validation of the eight genes signature in GSE72094 dataset. (A) KM plot for OS grouped by risk score in whole population. (B) Univariate cox analysis of OS. (C) Multivariate analysis of OS. (D) AUC of tROCs for OS at 1‐year, 3‐year, and 5‐year. (E) KM plot for OS grouped by risk score in validation cohort. (F) Univariate cox analysis of OS in validation cohort. (G) Multivariate cox analysis of OS in validation cohort. (H) AUC of tROCs for OS at 1‐year, 3‐year, and 5‐year in validation cohort. Abbreviations: KM plot, Kaplan‐Meier plot; OS, overall survival; TCGA, The Cancer Genome Atlas

These results were then validated in the external cohort, KM‐plot showed a consisted result as discovery cohort (Figure 3E), UCA indicated stage, KRAS status, and risk score were prognosis‐related (Figure 3F), stage and risk score were then proved to be independent risk factors for OS by MCA, and tROCs showed a max AUC of 0.7176205 for risk score at 1‐year (Figure 3G).

To further investigate underlying mechanism of this prognosis relevance, functional analysis was implemented. Differential‐expressed genes (DEGs) bewteen high‐ and low‐risk patients were identified (Figure 4A), and further KEGG analysis with DEGs mainly enriched in metabolic pathways, for instance steroid hormone biosynthesis, fat digestion, and absorption (Figure 4B). GO enrichment mainly enriched in ion transportation and leukotriene metabolism pathways (Figure 4C). Immuno‐infiltration status deconvolution by single sample gene set enrichment analysis (ssGSEA) (Table S4) showed high‐risk patients were associated with a decreased score of type I and II T helper cell, immature and activated dendritic cell, and effector memory CD8 T cell (Figures 4D and 4E); gene set variation analysis showed decreased score of antigen processing and presentation pathway in high‐risk patients, which is consistent with ssGSEA results (Figure 4F), and pathways related to glutathione metabolism, steroid biosynthesis, sphingolipid metabolism, and chemokine signaling were also varied in two groups.

FIGURE 4Functional analysis. (A) Volcano plot of differential expressed genes between high‐ and low‐risk patients. (B) Top 10 KEGG pathways enriched. (C) Top 10 GO pathways enriched. (D) ssGSEA score heatmap of immune infiltration in LUAD samples from TCGA. (E) ssGSEA score of 28 immune cells grouped by risk score. (F) GSVA score for KEGG pathways grouped by high and low risks. Only data with significance that is *p* < 0.05 were showed here due to space limitation. (G) Transcription factors of ferroptosis‐related genes predicted by RcisTarget. From left to right: Recovery curve of the gene‐set on the motif ranking; regulating network of TF; motif sequence diagram. More motifs could be seen in Figure S1. (H) Volcano plot of correlation between hub gene expression and drug response AUC value in LUAD cell lines. *p* < 0.05 and Pearson correlation coefficient > 0.4 were considered significantly correlated, and a negatively correlation means higher expression of this gene was correlated with better response (smaller response AUC value). *p < 0.05, **p < 0.01, ***p < 0.001. Abbreviations: AUC, area under curve; GO, gene ontology; GSVA, gene set variation analysis; KEGG: Kyoto Encyclopedia of Genes and Genomes; LUAD, lung adenocarcinoma; ssGSEA, single sample gene set enrichment analysis; TF, transcription factor

**Figure:**
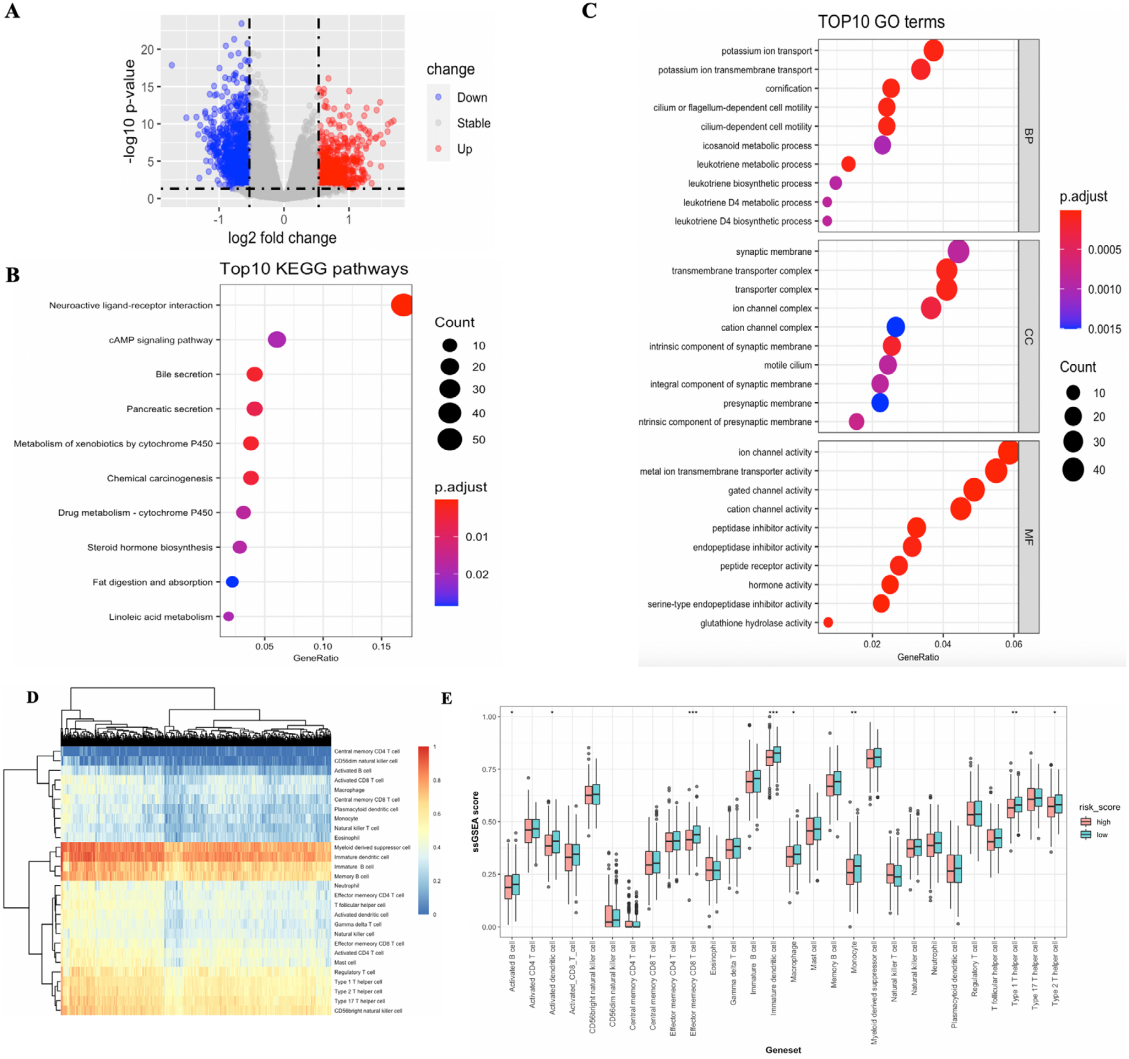

**Figure:**
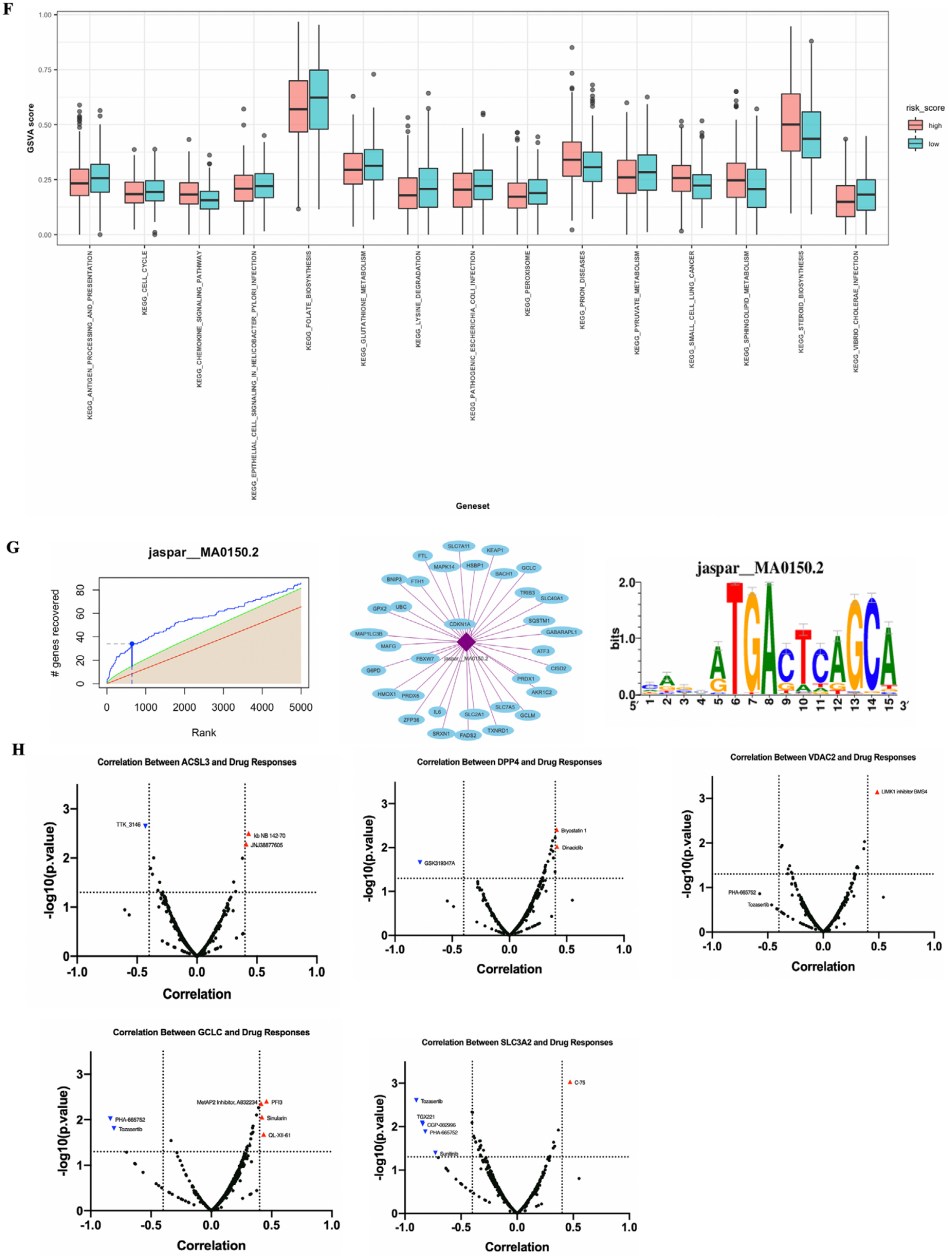

RcisTarget,^6^ an R package usually used in single‐cell transcriptome data, was then applied on 274 ferroptosis‐related genes to identify potential transcription factors (TFs). A total of 74 potential TFs were identified, and five TFs with maximum AUCs were annotated as Erythroid Derived 2 Like Protein 2, Nuclear Factor‐Erythroid 2, MAF BZIP Transcription Factor K, Activating Transcription Factor 4, and CCAAT Enhancer Binding Protein Gamma, respectively (Figures 4G and S3). Then we carried out integrated analysis of transcriptome data from cancer cell line encyclopedia and drug response data (response AUC value) from cancer therapeutics response portal respectively to identify potential candidate drugs for risky genes in our signature (Figure 4H). Interestingly, Tozasertib (an Aurora Kinase inhibitor) and PHA‐665752 (a c‐MET inhibitor) were found strongly correlated with both GCLC and SLC3A2 expression, as well as VDAC2, although not statistically significant (*p* > 0.05).

In conclusion, novel ferroptosis‐related prognosis‐related molecules for LUAD have been identified, a novel eight‐gene signature was constructed and validated, prove to have good capacity in predicting OS in LUAD. Functional analysis showed that immune‐related pathways may be involved in the regulation of ferroptosis. Potential TFs regulating ferroptosis and several candidate drugs targeted the risk gene were identified. Our findings could provide hints for further study and warrant further research on ferroptosis as a functional and therapeutic target in LUAD.

## ETHICS APPROVAL AND CONSENT TO PARTICIPATE

All the data involved were publicly available, so the ethics approval was waived. The authors are accountable for all aspects of the work in ensuring that questions related to the accuracy or integrity of any part of the work are appropriately investigated and resolved.

## CONFLICT OF INTEREST

The authors declare no conflict of interest.

## DATA AVAILABILITY STATEMENT

All the data involved were publicly available on https://cancergenome.nih.gov/ and https://www.ncbi.nlm.nih.gov.

## Supporting information

Figure S1 (A‐E) KM‐plot for OS in different subpopulation of TCGA patients

Figure S2 Expression pattern of modeling genes

Figure S3 Representative motifs enriched by RcisTarget

Supplementary materials including Table S1‐S4 and Figure S1‐12 were uploaded separately due to space limitation.

Table S1 Full List of Ferroptosis‐Related Genes

Table S2 Summary of eight genes selected for modeling

Table S3 Clinical characteristics of patients in low‐ and high‐risk group

Table S4 Immune‐Infiltration gene set for ssGSEA
