# Supplementary figures and images for: Clinical and functional significance of a novel ferroptosis‐related prognosis signature in lung adenocarcinoma
Source: Clin Transl Med. 2021 Mar 17;11(3):e364. doi: 10.1002/ctm2.364 (PMC7968124; doi:10.1002/ctm2.364)

**Fig S1** A-E. KM-plot for OS in different subpopulation of TCGA patients


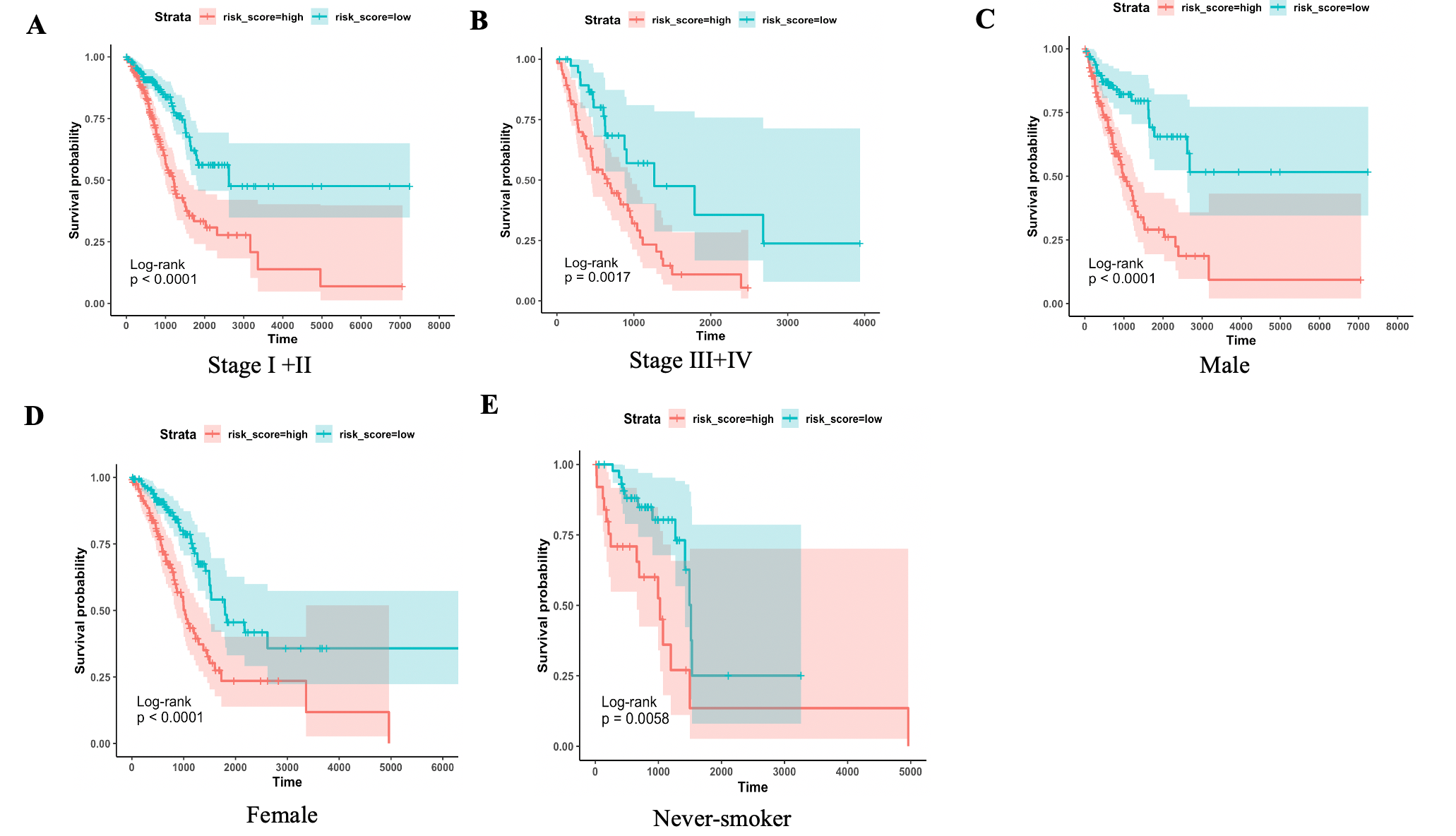

Supplement: Supplementary file 1 — Figure S1 (A‐E) KM‐plot for OS in different subpopulation of TCGA patients [file CTM2-11-e364-s005.docx]

**Fig S3.** Representative motifs enriched by RcisTarget


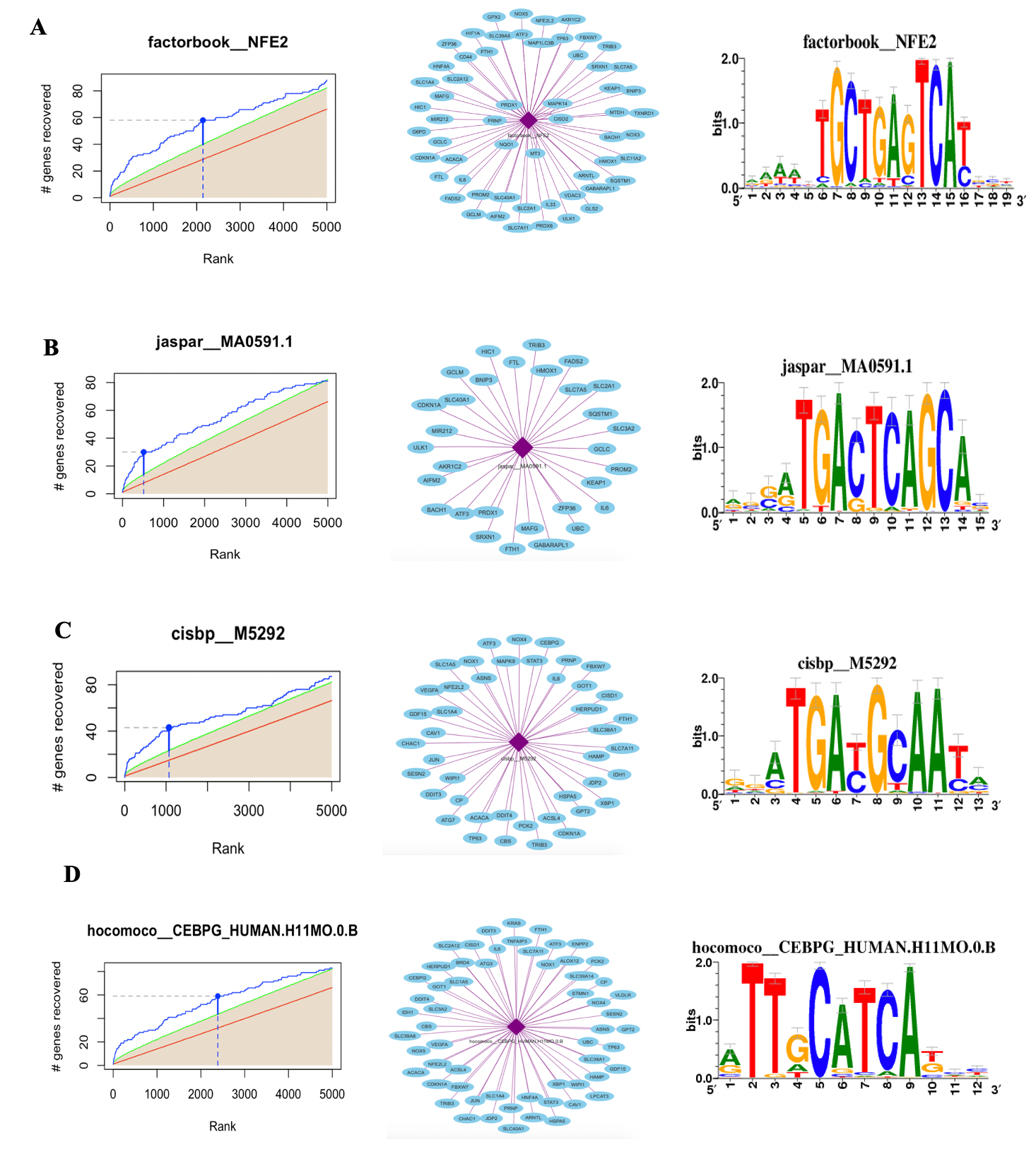

Supplement: Supplementary file 3 — Figure S3 Representative motifs enriched by RcisTarget [file CTM2-11-e364-s004.docx]
