# Supplementary material for: Clinical and functional significance of a novel ferroptosis‐related prognosis signature in lung adenocarcinoma
Source: Clin Transl Med. 2021 Mar 17;11(3):e364. doi: 10.1002/ctm2.364 (PMC7968124; doi:10.1002/ctm2.364)
Supplement: Supplementary file 2 — Figure S2 Expression pattern of modeling genes [file CTM2-11-e364-s001.docx]

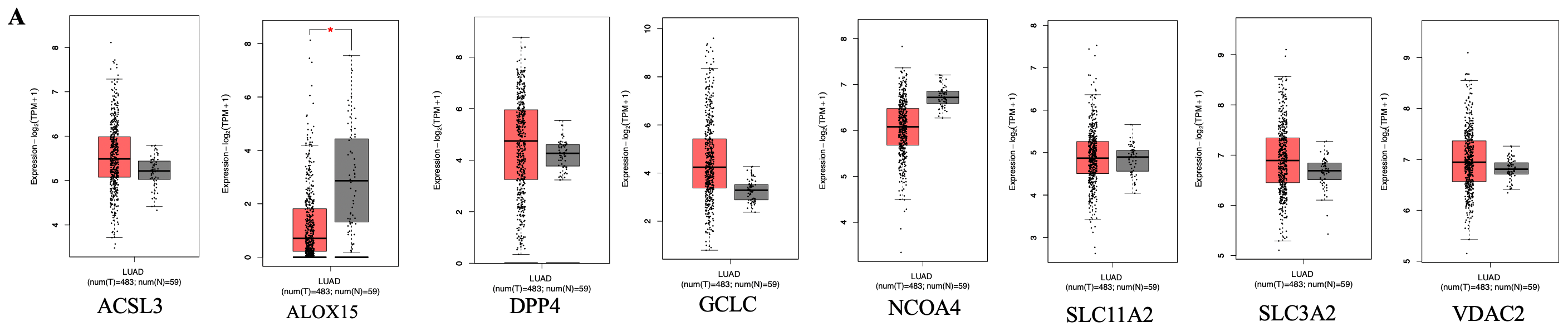


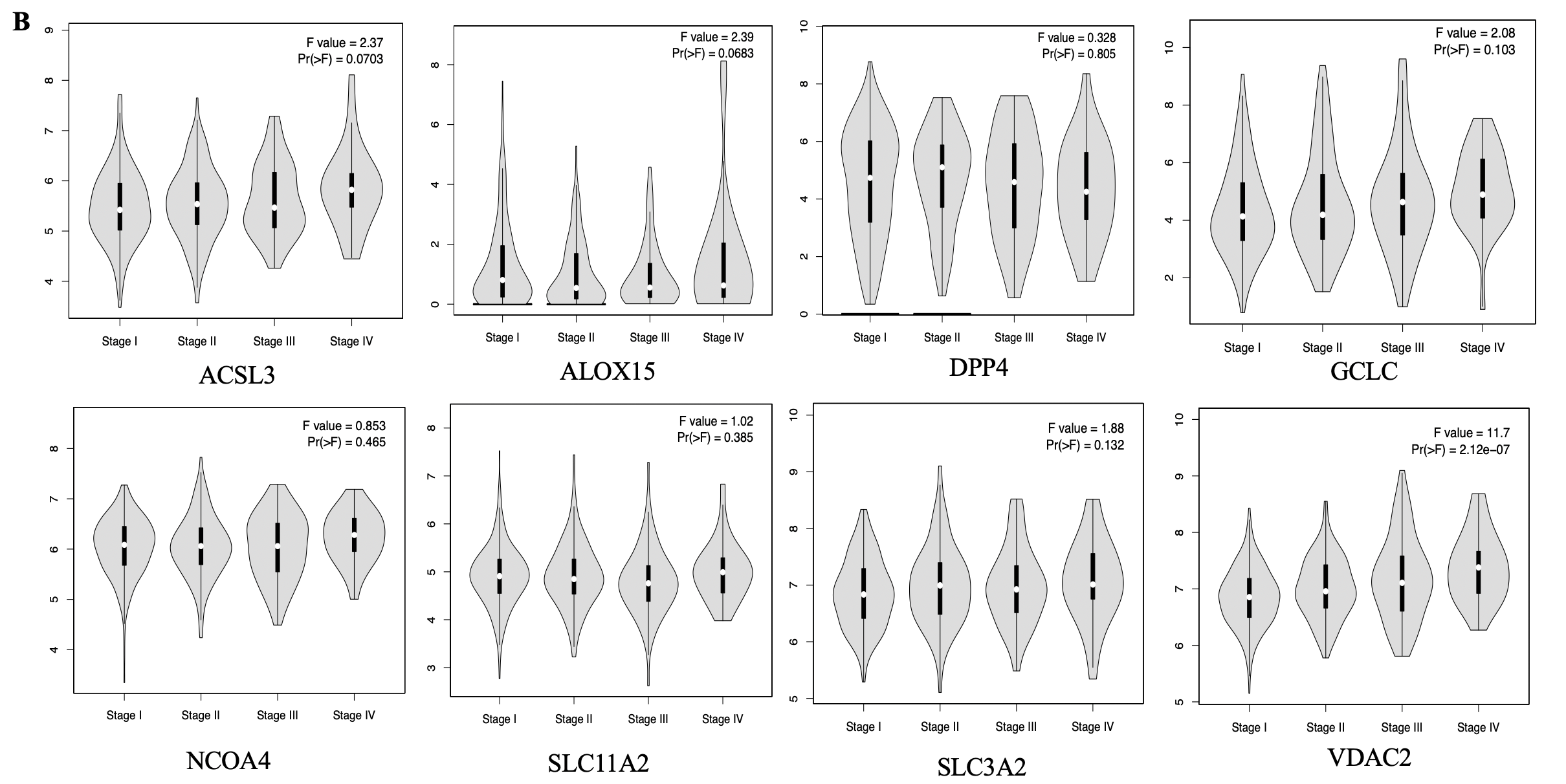


**Figure S2** A. Expression level of modeling genes obtained from Gepia-2. B. Expression by pathological stage. * p<0.05; ** p<0.01; *** p<0.001; ns, not significant.
