# Supplementary material for: Clinical and functional significance of a novel ferroptosis‐related prognosis signature in lung adenocarcinoma
Source: Clin Transl Med. 2021 Mar 17;11(3):e364. doi: 10.1002/ctm2.364 (PMC7968124; doi:10.1002/ctm2.364)
Supplement: Supplementary file 4 — Supplementary materials including Table S1‐S4 and Figure S1‐12 were uploaded separately due to space limitation. [file CTM2-11-e364-s002.docx]

**Methods**

*Study design and data accession*

Level 3 RNA-seq and somatic mutation data as well as clinical information of LUAD cases were obtained from The Cancer Genome Atlas (TCGA) database (<https://tcga-data.nci.nih.gov/tcga/>) as discovery cohort, contained a total of 526 tumor, and 59 normal lung tissues, 21 tumor tissues were excluded due to lack of survival or stage information. GSE72094 dataset were downloaded from Gene Expression Omnibus (GEO) (<https://www.ncbi.nlm.nih.gov/geo/>), 44 cases were excluded due to lack of survival information, and a total of 398 cases with RNA-seq, hotspot genes mutation and clinical information were used as an independent validation cohort. Ferroptosis-related genes were selected from on-line database (<http://www.zhounan.org/ferrdb/>) and literature review,^13-25^ full gene-list could be obtained from Supplementary Table S1.

*Construction and validation of prognosis signature*

The “limma” R package was used for differentially expressed genes (DEGs) identifying with false discovery rate (FDR)<0.05. Univariate cox regression was carried out to identify prognosis-related genes, least absolute shrinkage and selection operator (LASSO) was carried out with “glmnet” R package based on the result of univariate cox regression to minimize the risk of overfitting, tenfold cross-validation was performed to identify the optimal λ value, then Proportional Hazards Assumption (PH) test was performed with “survival” R package to ensure that all the factors were time-independent, p<0.05 was considered unfit for Cox model. Variance Inflation Factor (VIF) was then calculated with “rms” R package, factors with VIF>2 was considered collinear and will be excluded. Cox model was used to build the prognosis signature and risk score was calculated for each patient with “survival” R package as follows: Risk score = Coefficient _gene-1_ * expression level of gene-1 + Coefficient _gene-2_ * expression level of gene-2 +.... + Coefficient _gene-n_ * expression level of gene-n. Patients were then divided into high-risk or low-risk group according to the mean of risk score. Univariate cox regression, multivariate regression, KM curve and tROC were used to evaluate the prognosis capacity of the signature with “survival”, “survminer”, “survivalROC” R packages respectively.

*Functional Analysis*

“Rcistarget” R package^27^ was used to identify potential TFs, Normalized enrichment score of the motif in the gene-set (NES) was calculated by AUC, an NES>0.3 is considered significant. RcisTarget identifies transcription factor binding motifs (TFBS) over-represented on a gene list. In a first step, RcisTarget selects DNA motifs that are significantly over-represented in the surroundings of the transcription start site (TSS) of the genes in the gene-set. This is achieved by using a database that contains genome-wide cross-species rankings for each motif. The motifs that are then annotated to TFs and those that have a high Normalized Enrichment Score (NES) are retained. Finally, for each motif and gene-set, RcisTarget predicts the candidate target genes (<https://anaconda.org/bioconda/bioconductor-rcistarget>). GO and KEGG enrichment analysis were carried out with “clusterProfiler” R package based on the DEGs (FDR<0.05, |log2 Fold Change (log2FC) ≥0.3|) between high and low risk patients, ssGSEA was performed to investigate the immune infiltration status, the gene-set for ssGSEA was provided in Supplementary Table S2, and GSVA was also performed, GSVA gene-set was downloaded from Molecular Signatures Database v7.2 (<https://www.gsea-msigdb.org/gsea/index.jsp>). ssGSEA and GSVA were performed with “GSVA” R package, ssGSEA and GSVA scores were compared between high and low risk groups with “limma” package. Next, we obtained gene expression data of cancer cell lines from CCLE and drug response data from CTRP, 50 LUAD cell lines contained both type of data were extracted, with a total of 447 drugs were analysis. Pearson correlation coefficient of drug response AUC (a smaller AUC is correlated with higher sensitivity and vice versa) and expression level of risky genes (coefficient>0) in our signature was calculated in R, p<0.05 and |correlation coefficient|>0.04 was considered significant.

*Statistical Analysis*

Continuous data were compared with t-test and proportions were compared with Chi-squared test. Paired-data analysis were using Wilcox test. Log-rank test was used to test the significance of KM curves. All the statistical analysis were performed in R version 4.0.2. Graphs are drawing with R version 4.0.2 or GraphPad prism version 8.3.1.
