# Supplementary material for: Clinical and functional significance of a novel ferroptosis‐related prognosis signature in lung adenocarcinoma
Source: Clin Transl Med. 2021 Mar 17;11(3):e364. doi: 10.1002/ctm2.364 (PMC7968124; doi:10.1002/ctm2.364)
Supplement: Supplementary file 6 — Table S2 Summary of eight genes selected for modeling [file CTM2-11-e364-s003.docx]

| **Gene symbol** | **Ensembl ID** | **Gene name** | **Coefficient** | | **P value for PH test** | | **VIF** | **Known function (from Entrez Gene Summary)** |
| --- | --- | --- | --- | --- | --- | --- | --- | --- |
| ACSL3 | ENSG00000123983 | Acyl-CoA Synthetase Long Chain Family Member 3 | | 3.40 | | 0.30 | 1.28 | The protein encoded by this gene is an isozyme of the long-chain fatty-acid-coenzyme A ligase family. Although differing in substrate specificity, subcellular localization, and tissue distribution, all isozymes of this family convert free long-chain fatty acids into fatty acyl-CoA esters, and thereby play a key role in lipid biosynthesis and fatty acid degradation. This isozyme is highly expressed in brain, and preferentially utilizes myristate, arachidonate, and eicosapentaenoate as substrates. The amino acid sequence of this isozyme is 92% identical to that of rat homolog. Two transcript variants encoding the same protein have been found for this gene ^[1]^. |
| ALOX15 | ENSG00000161905 | Arachidonate 15-Lipoxygenase | | -0.32 | | 0.46 | 1.12 | This gene encodes a member of the lipoxygenase family of proteins. The encoded enzyme acts on various polyunsaturated fatty acid substrates to generate various bioactive lipid mediators such as eicosanoids, hepoxilins, lipoxins, and other molecules. The encoded enzyme and its reaction products have been shown to regulate inflammation and immunity. Multiple pseudogenes of this gene have been identified in the human genome ^[2]^. |
| DPP4 | ENSG00000197635 | Dipeptidyl Peptidase 4 | | 0.16 | | 0.052 | 1.42 | The DPP4 gene encodes dipeptidyl peptidase 4, which is identical to adenosine deaminase complexing protein-2, and to the T-cell activation antigen CD26. It is an intrinsic type II transmembrane glycoprotein and a serine exopeptidase that cleaves X-proline dipeptides from the N-terminus of polypeptides. Dipeptidyl peptidase 4 is highly involved in glucose and insulin metabolism, as well as in immune regulation. This protein was shown to be a functional receptor for Middle East respiratory syndrome coronavirus (MERS-CoV), and protein modeling suggests that it may play a similar role with SARS-CoV-2, the virus responsible for COVID-19 ^[3]^. |
| GCLC | ENSG00000001084 | Glutamate-Cysteine Ligase Catalytic Subunit | | 0.14 | | 0.60 | 1.25 | Glutamate-cysteine ligase, also known as gamma-glutamylcysteine synthetase is the first rate-limiting enzyme of glutathione synthesis. The enzyme consists of two subunits, a heavy catalytic subunit and a light regulatory subunit. This locus encodes the catalytic subunit, while the regulatory subunit is derived from a different gene located on chromosome 1p22-p21. Mutations at this locus have been associated with hemolytic anemia due to deficiency of gamma-glutamylcysteine synthetase and susceptibility to myocardial infarction ^[4]^. |
| NCOA4 | ENSG00000266412 | Nuclear Receptor Coactivator 4 | | -3.93 | | 0.14 | 1.57 | This gene encodes an androgen receptor coactivator. The encoded protein interacts with the androgen receptor in a ligand-dependent manner to enhance its transcriptional activity. Chromosomal translocations between this gene and the ret tyrosine kinase gene, also located on chromosome 10, have been associated with papillary thyroid carcinoma. Alternatively spliced transcript variants have been described. Pseudogenes are present on chromosomes 4, 5, 10, and 14 ^[5]^. |
| SLC11A2 | ENSG00000110911 | Solute Carrier Family 11 Member 2 | | -3.00 | | 0.49 | 1.17 | This gene encodes a member of the solute carrier family 11 protein family. The product of this gene transports divalent metals and is involved in iron absorption. Mutations in this gene are associated with hypochromic microcytic anemia with iron overload. A related solute carrier family 11 protein gene is located on chromosome 2. Multiple transcript variants encoding different isoforms have been found for this gene ^[6]^. |
| SLC3A2 | ENSG00000168003 | Solute Carrier Family 3 Member 2 | | 0.05 | | 0.073 | 1.58 | This gene is a member of the solute carrier family and encodes a cell surface, transmembrane protein. The protein exists as the heavy chain of a heterodimer, covalently bound through disulfide bonds to one of several possible light chains. The encoded transporter plays a role in regulation of intracellular calcium levels and transports L-type amino acids. Alternatively spliced transcript variants, encoding different isoforms, have been characterized ^[7]^. |
| VDAC2 | ENSG00000165637 | Voltage Dependent Anion Channel 2 | | 2.45 | | 0.76 | 1.64 | This gene encodes a member of the voltage-dependent anion channel pore-forming family of proteins that are considered the main pathway for metabolite diffusion across the mitochondrial outer membrane. The encoded protein is also thought to be involved in the mitochondrial apoptotic pathway via regulation of BCL2-antagonist/killer 1 protein activity. Pseudogenes have been identified on chromosomes 1, 2, 12 and 21, and alternative splicing results in multiple transcript variants ^[8]^. |

Table S2. Summary of 8 genes selected for modeling

**References**

1. Entrez Gene Summary for ACSL3. Retrieved from: https://www.ncbi.nlm.nih.gov/gene/2181.

2. Entrez Gene Summary for ALOX15. Retrieved from: https://www.ncbi.nlm.nih.gov/gene/246.

3. Entrez Gene Summary for DPP4. Retrieved from: https://www.ncbi.nlm.nih.gov/gene/1803.

4. Entrez Gene Summary for GCLC. Retrieved from: https://www.ncbi.nlm.nih.gov/gene/2729.

5. Entrez Gene Summary for NCOA4. Retrieved from: https://www.ncbi.nlm.nih.gov/gene/8031.

6. Entrez Gene Summary for SLC11A2. Retrieved from: https://www.ncbi.nlm.nih.gov/gene/4891.

7. Entrez Gene Summary for SLC3A2. Retrieved from: https://www.ncbi.nlm.nih.gov/gene/6520.

8. Entrez Gene Summary for VDAC2. Retrieved from: https://www.ncbi.nlm.nih.gov/gene/7417.

Footnote: PH test: proportional hazards assumption test; VIF: Variance Inflation Factor.
