# Supplementary material for: Clinical and functional significance of a novel ferroptosis‐related prognosis signature in lung adenocarcinoma
Source: Clin Transl Med. 2021 Mar 17;11(3):e364. doi: 10.1002/ctm2.364 (PMC7968124; doi:10.1002/ctm2.364)
Supplement: Supplementary file 7 — Table S3 Clinical characteristics of patients in low‐ and high‐risk group [file CTM2-11-e364-s008.docx]

| Characteristic | TCGA-LUAD | | Chi-square value | p-value | GSE72049 | | Chi-square value | p-value |
| --- | --- | --- | --- | --- | --- | --- | --- | --- |
|  | Low risk  (n=252) | High risk  (n=253) |  |  | Low risk  (n=199) | High risk  (n=199) |  |  |
|  | n (%) | n (%) |  |  | n (%) | n (%) |  |  |
| Age(years) |  |  | 0.410 | 0.522 |  |  | 5.187 | 0.023* |
| ≤60 | 76 (30.16%) | 83 (32.81%) |  |  | 25 (12.56%) | 42 (21.11%) |  |  |
| >60 | 176 (69.84%) | 170 (67.19%) |  |  | 174 (87.44%) | 157 (78.90%) |  |  |
| Gender |  |  | 0.109 | 0.741 |  |  | 0.163 | 0.686 |
| Female | 154 (61.11%) | 119 (47.04%) |  |  | 113 (56.78%) | 109 (54.78%) |  |  |
| Male | 98 (38.89%) | 134 (52.96%) |  |  | 86 (43.22%) | 90 (45.23%) |  |  |
| Smoking |  |  | 7.017 | 0.030* |  |  | 1.979 | 0.372 |
| Former or current smoker | 201 (79.76%) | 219 (86.56%) |  |  | 156 (78.39%) | 144 (72.36%) |  |  |
| Never-smoker | 46 (18.25%) | 26 (10.28%) |  |  | 14 (7.04%) | 17 (8.54%) |  |  |
| Unknown | 5  (1.98%) | 8  (3.16%) |  |  | 29 (14.52%) | 38 (19.10%) |  |  |
| Stage |  |  | 7.390 | 0.007** |  |  | 3.165 | 0.205 |
| I+II | 212 (84.13%) | 188 (74.30%) |  |  | 160 (80.40%) | 145 (72.86%) |  |  |
| III+IV | 40 (15.87%) | 65  (25.69%) |  |  | 37 (18.59%) | 51 (25.63%) |  |  |
| Unknown | 0 | 0 |  |  | 2  (1.01%) | 3  (1.51%) |  |  |
| KRAS status |  |  | 6.020 | 0.014* |  |  | 9.297 | 0.002** |
| Mutation | 38 (15.08%) | 60 (23.72%) |  |  | 55 (27.64%) | 84 (42.21%) |  |  |
| Wild | 214 (84.92%) | 193 (76.59%) |  |  | 144 (72.36%) | 115 (57.79%) |  |  |
| EGFR status |  |  | 0.473 | 0.492 |  |  | 3.290 | 0.070 |
| Mutation | 21  (8.33%) | 17  (6.72%) |  |  | 26 (13.07%) | 15 (7.54%) |  |  |
| Wild | 231 (91.67%) | 236 (93.28%) |  |  | 173 (86.93%) | 184 (92.46%) |  |  |
| TP53 status |  |  | 16.050 | 0.000*** |  |  | 7.211 | 0.007** |
| Mutation | 73 (28.97%) | 117 (46.25%) |  |  | 37 (18.59%) | 60 (30.15%) |  |  |
| Wild | 179 (71.03%) | 136 (53.75%) |  |  | 162 (81.41%) | 139 (69.85%) |  |  |

Table S3. Clinical characteristics of patients in low and high-risk group

Footnote: * p<0.05; ** p<0.01; *** p<0.001
